# Two-color synchrotron X-ray spectroscopy based on transverse resonance island buckets

**DOI:** 10.1038/s41598-022-19100-z

**Published:** 2022-09-01

**Authors:** K. Holldack, C. Schüßler-Langeheine, N. Pontius, T. Kachel, P. Baumgärtel, Y. W. Windsor, D. Zahn, P. Goslawski, M. Koopmans, M. Ries

**Affiliations:** 1 Helmholtz-Zentrum Berlin für Materialien und Energie GmbH, Albert-Einstein-Str. 15, 12489 Berlin, Germany; 2 Fritz Haber Institute of the Max Planck Society, Faradayweg 4-6, 14195 Berlin, Germany

**Keywords:** Physics, Applied physics

## Abstract

We report on a novel multi-color method of X-ray spectroscopy at a Synchrotron radiation source that uses two simultaneously filled electron orbits in an electron storage ring to generate multiple soft or tender X-ray beams of different wavelength. To establish the second orbit, we use nonlinear beam dynamics in the so called TRIBs—transverse resonance island buckets—mode of the BESSY II storage ring, where a second electron orbit winds around the regular one leading to transversely separated source points. X-ray beams of multiple colors are generated by imaging the individual source points via different pathways through a monochromator. The particular colors can be varied by changing the traversal electron beam positions through storage-ring parameters and/or via the monochromator dispersion. As a proof of principle, X-ray absorption spectroscopy is performed on thin Fe films in transmission as well as a scanning transmission measurement on a Fe_3_GeTe_2_ sample of inhomogeneous thickness normalizing resonant signals with the pre-edge intensity. Using the extraordinary pointing fidelity of successive X-ray macro-pulses arriving at MHz repetition rates, a detection of tiny contrasts in diluted systems, contrast enhancement in X-ray microscopy as well as fast dynamics studies come into reach.

## Introduction

Resonant X-ray spectroscopy and microscopy are indispensable tools in modern materials science for quantum and energy materials, in life- and environmental sciences as well as metrology and they will be boosted in near future by the new modern 4th generation light sources^1^ coming to life. To deal with contributions to X-ray spectra that are not sample related like energy dependence of the photon flux or the detection efficiency and in particular fluctuations in the x-ray beam intensity and pointing, usually a reference value of the photon flux is recorded. Ideally this is done as close to the sample as possible, t.e. without additional modification by further optical components and with a well calibrated detector. Moreover, since fluctuations in x-ray beams occur over a wide range of frequencies, the reference signal should best be measured simultaneously of quasi-simultaneously and on the shortest possible timescale. Common methods for soft x-ray experiments are (i) DC measurements of photocurrents from upstream mirrors, or (ii) upstream gold-meshes or (iii) a part of the beam (e.g. other diffraction order) is reflected to a separate detector providing a reference signal. Usually, a reference signal of the same photon energy is being used, but for some experiments a reference signal of different x-ray energy is preferable. In the present paper we show how to use an electron storage ring mode in which the electrons circulate in an orbit that closes only after three revolutions together with a direct imaging monochromator to provide time-separated X-ray signals on one single detector that alternate with MHz frequency between three different x-ray energies separated by several eV. Apart from the energy offset the different x-ray signals are fully equivalent and can serve as reference signals for each other. We demonstrate this here in a proof-of-principle experiment using the near-edge absorption of a thin film of the dichalcogenide Fe_3_GeTe_2_ at the Fe L_3,2_ edges by measuring transmission spectra with monochromatic macropulses emitted from electrons of successive turns in the TRIBs mode^2–5^ at single island population^3^ (SIP) at BESSY II.

We demonstrate how to use this for solving a contrast problem in scanning X-ray transmission spectroscopy from a challenging sample with variable thickness: With an off-resonance reference signal we can determine the local sample thickness quasi simultaneously and independently from the spectroscopic information. As the non-resonant reference beam is produced by the same electrons as the resonant beam, it contains the information of fluctuations in the storage ring down to sub-µs time scales and can be used to normalize these out. Such MHz normalization can improve the signal-to-noise ratio by orders of magnitude because the pointing stability of electron- and photon beams on micro-second time scales is up to 5 orders of magnitude better than for comparing signal and reference in the second or minute regime—the beam “stands still” between successive turns but jitters much more in the low frequency band owing to vibrations and other sources^6^. Furthermore, the non-resonant reference beam co-propagates with the resonance beam allowing for high flexibility in the experimental design. Both beams can generally be detected with the same detector separating the resonant and non-resonant signal via their arrival time on the detector. All this enables high contrast and unprecedented sensitivity for dilute species and for samples where a reference signal *I*_*o*_ is hard to get.

Our approach is to convert a transversal offset in the electron beam position into an energy shift. We start by estimating the size of the effect. In the soft and tender X-ray region, grazing incidence grating monochromators^7^ act as workhorses that image the source point in the storage ring along the dispersion plane onto an exit slit. We used the planar grating monochromator (PGM), here at the PM3 beamline at BESSY II^8^. We begin by estimating how much the photon energy, *E*, on the sample behind the slit shifts when the source point is set transversally off in the dispersion plane.

**Figure 1:**
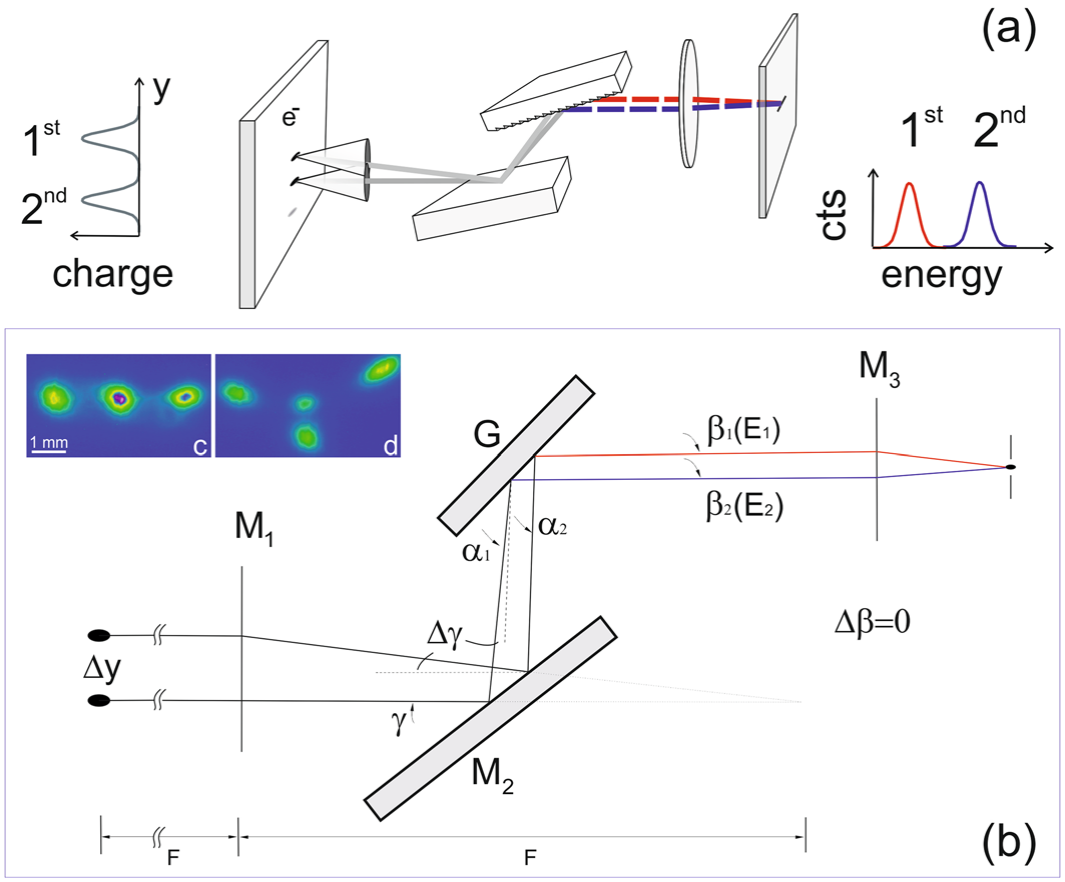
Sketches of the dispersion performed by a collimating plane grating monochromator and source point images from a dipole (**a**, **b**) which lay flat at a decoupled machine (**c**) and are displaced in the y-plane (**d**) forced by different skew-quadrupole settings. Charge displacement by Δy along the dispersion plane in (**b**) translates into a spectral separation ΔE behind the exit slit of direct imaging monochromators (e.g. a plane grating monochromator) leading to a change in color of light that hits the sample (here in transmission geometry). Here, γ, α denote grazing incidence angles on the pre-mirror M_2_ and the grating, respectively. Light of the same energy from a displaced bunch is blocked by the exit slit but rays of different energy from the two sources may only pass if they follow the same angle β_1_ = β_2_, the grazing angle of the rays reflected by the grating.

The angular dispersion relation d*E*/d*y* for a vertical source displacement d*y* (see Fig. 1) for a collimating PGM monochromator is derived (see Supplementary Note 1) according to Petersens^8^ and Follath’s^9^ work for grazing angles α on the grating:

1 $$ \frac{d}{dy} = \frac{{^{ 2} }}{GhcF}\sin \alpha $$

where *hc* is the product of Planck’s constant *h* and the speed of light c, *F* is the focal length of the pre-mirror and *G* the groove density of the blazed grating (here *G* = 1221 l/mm). PGMs at BESSY II are operated with light collimated along the dispersion plane by a toroidal pre-mirror under fix focus conditions^8,9^ at the ratio c_ff_ = sin β/sin α (see Fig. 1). For the grazing incidence angle α on the grating vs. photon energy E follows:

2 $$ \alpha \left( {E,c_{ff} ,G} \right) = \cos^{ - 1} \left( {\frac{hcG}{E{\left( {1 - c_{ff}^{2} } \right)}} + \sqrt {1 + \left( {\frac{{c_{ff} hcG}}{{\left( {c_{ff}^{2} - 1} \right)E}}} \right)^{2} } } \right). $$

We note in Eq. (1) with (2) that the dispersion depends on the *c*_*ff*_ value and the groove density of the grating, both useful to tune the desired energy shift ΔE at a fixed accelerator setting by a few eV while preserving resolution and flux. For E = 700 eV and c_*ff*_ = 2.25 (standard value) Eq. (1) yields dE/dy = 0.81 eV/mm (sin α = 0.032, α = 1.84°). Given that using TRIBs population can displace the electron beam by up to 10 mm (see Methods section), this would enable tunable energy shifts of up to 10 eV. Using another grating with G = 600 l/mm would double the energy shift according to Eq. (1) likewise done by changing c_ff_ as revealed by Eq. (1) and (2). Results from detailed raytracing with all realistic values for the PM3 are shown in Supplementary Fig. S1-2 and Supplementary Tables S1-3. There is a very good agreement between the simple Eq. (1) and raytracing with the experimental dE/dy values being slightly higher, which is likely given by the fact that existing vertical angular changes y’ between beams from successive turns was ignored in both by Eqs. (1) and (2) and by raytracing.

Encouraged by the simple estimates from Eq. (1) we used BESSY II in a special TRIBs mode with single island population featuring an orbit closing only after three turns. In a x–y-decoupled low emittance mode, the source points are separated horizontally by a few mm (see Supplementary Fig. S3-4). For a monochromator with horizontal dispersion plane, this horizontal separation could directly be converted into an energy shift. Unfortunately, all 3rd generation storage rings are designed for small vertical emittance by operating at small coupling of 1–2% making source points usually vertically flat ellipses. To achieve high resolution in monochromators, all dispersion planes of monochromators are in vertical direction and there is no easy way to change this. Consequently, for our test experiment at the PM3 monochromator, with a vertical dispersion plane, we converted the horizontal separation of the source points into a vertical one. To this end, a vertical turn-by-turn offset of the source points was set by introducing a skew-quadrupole component that increases the x–y coupling of the storage ring setting (see Methods and Fig. 1b). This “coupled” operation causes the source points for different turns to be also vertically separated. This solution is good enough to demonstrate the suitability of our approach but implies an unwanted horizontal offset of the beam positions on the sample. As mentioned before, with a horizontally dispersion monochromator and “uncoupled” operation, the different beams were neatly separated only along the dispersive direction and would all reach the same focus spot on the sample. Particularly at 4th generation storage rings with “round” beams, i.e., with low emittance in both directions, a horizontal dispersion plane monochromator with high energy resolution is readily feasible.

## Results and discussion

**Figure 2:**
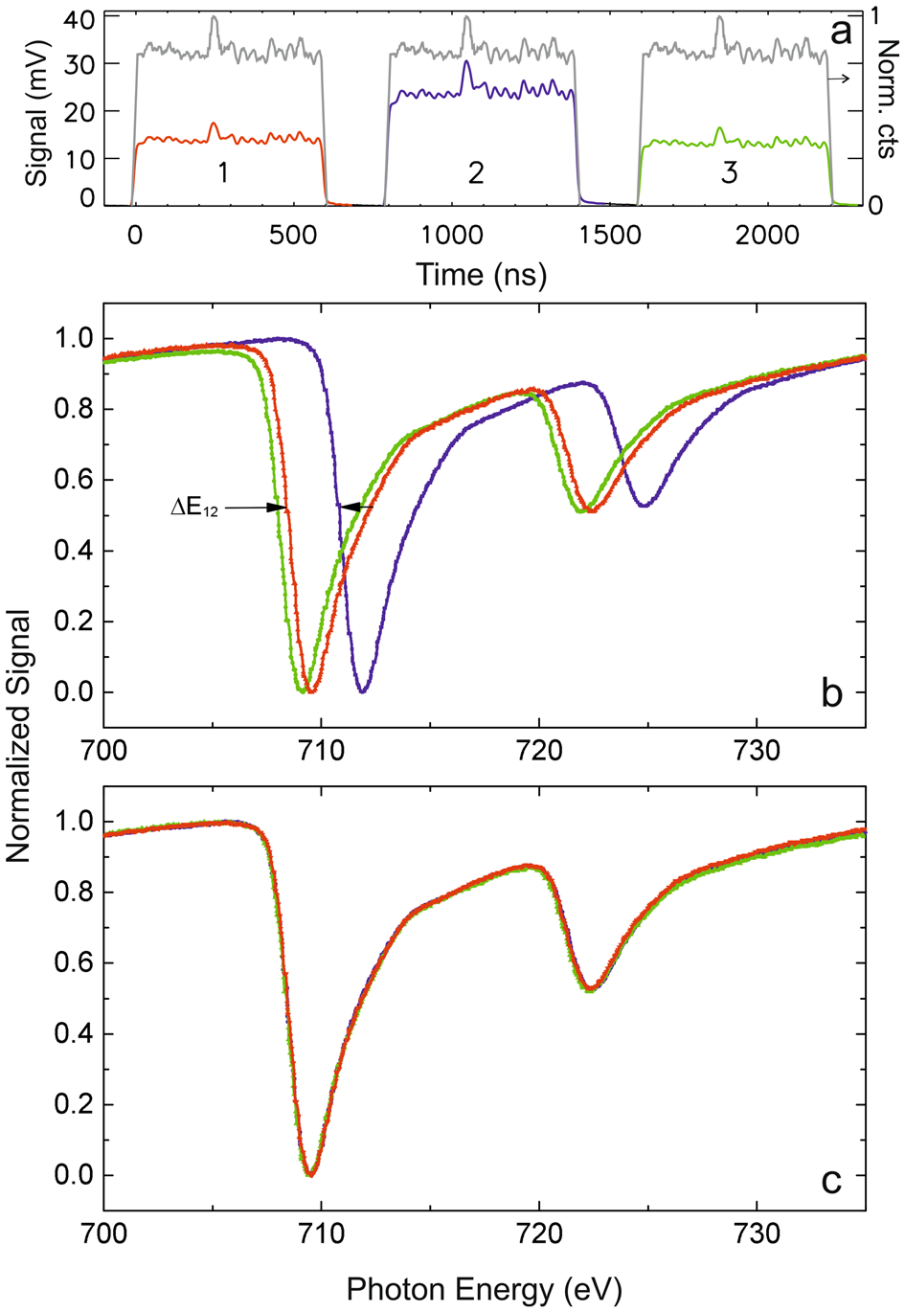
Signals and spectra from a Fe thin film sample using TRIBs simultaneously measured from successive macro pulses. (**a**) APD signal behind the sample revealing the difference of X-ray absorption at 709 eV versus time after the triple turn trigger event. A reference signal from the BESSY II fill-pattern APD detector for the 3 turns is also plotted (grey). (**b**) Normalized APD signals measured in parallel from the macropulses #1 (red), #2 (blue) and #3 (green) during fast scans (300 ms/point) of the photon energy behind the Fe sample in transmission for the TRIBs mode at single island population. Since the time resolution is about 5 ns, individual electron bunches (2 ns separation) are not resolved. (**c**) The same three spectra shift-corrected revealing no change in spectral shape using light emitted from different islands.

To detect a spectral shift expected from Eq. (1), we filled 300 out of 400 possible bunches of the regular orbit leading to a pulse train of 600 ns separated by 200 ns dark gap. The signal transmitted through the sample was measured simultaneously for three successive revolutions of the electron beam in the island orbit producing a temporal pattern like the gray line in Fig. 2a: The electron beam takes three round trips before its orbit is closed. Every round trip the same bunch produces an X-ray signal of equal intensity. The temporal structure of the X-ray macro pulses corresponds to the filling pattern of electron bunches in the storage ring. When we place an iron transmission sample in the beam and tune the monochromator to 709 eV photon energy, i.e., to the transmission minimum on the “white line” of the Fe *L*_3_ resonance, a temporal pattern as denoted by the colored curves in Fig. 2a is obtained. We now see the signal from the overall fill pattern with different intensity for the individual round trips. The reason for the different intensities becomes clear when we scan the monochromator to record the full transmission spectrum across the L_3_ and L_2_ edges in Fig. 2b. As expected, we see three different iron absorption spectra for x-rays emitted during different round trips. For the nominal monochromator setting of 709 eV used for the trace in Fig. 2a, the transmitted intensity of the signal from the 1st turn is weak, because the x-rays from this turn have a photon energy close to the resonant absorption maximum. X-rays from the 2nd turn are blue shifted as one needs a higher nominal monochromator energy to reach the absorption maximum; in the scan of Fig. 2a their photon energy was below the absorption threshold so that less signal is absorbed and higher intensities are transmitted through the sample. On the third turn, the photon energy is again near the absorption maximum.

The traces from the different turns are in fact identical in intensity and shape, except for their different photon energy; this shows Fig. 2c. It was generated by shifting the 2nd turn by ΔE = − 2.72 eV and the 3rd turn by ΔE = − 0.42 eV. The spectra shifted such are essentially identical in intensity and energy resolution, which makes the signals from different turns well suited to reference one against the other. A corresponding close-up of the L_3_ region for different (coupled) accelerator settings is also depicted in Supplementary Fig. S4 compared to a near zero vertical displacement in a decoupled machine as reference.

**Figure 3:**
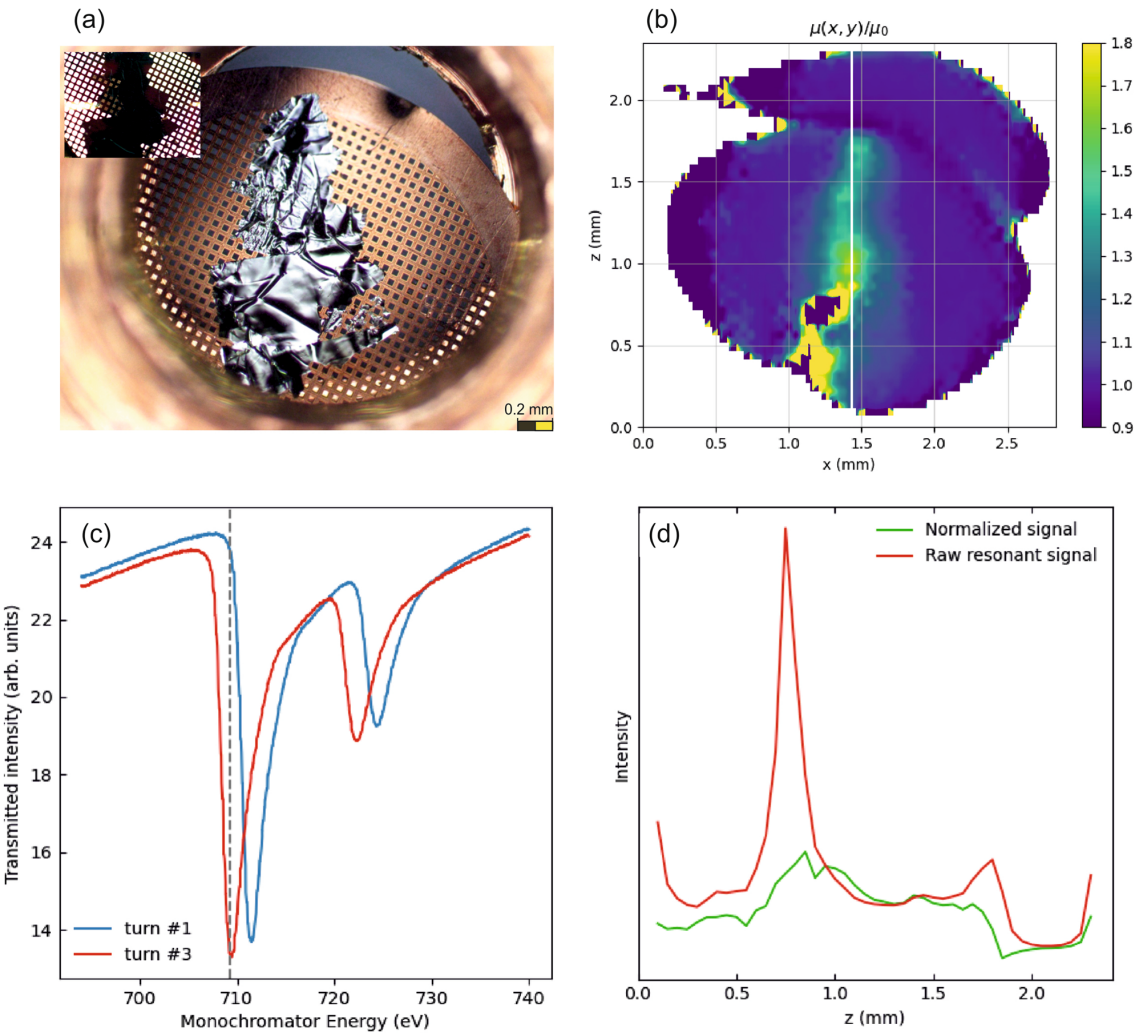
Images, spectra and analysis from a dichalcogenide Fe_3_GeTe_2_ thin film sample. (**a**) Visible light microscope image of the free standing thin film in reflection and a close up in transmission in the inset. (**b**) A transmission X-ray image as normalized turn by turn (turn #3/turn#1) at 709 eV. (**c**) Corresponding absorption spectra integrated over the full field across the Fe L-edges. (**d**) Raw intensity signal behind the sample at 709 eV along the vertical white line of the full map in (**b**) without (red) and after (green) turn-by-turn normalization (#1/#3) that eliminates the thickness contrast according to Lambert–Beer’s law, which fully dominates the raw signal (red). For detailed analysis of the maps and the line scan see Supplementary Note 2.

We will now make use of this multi-color beam to map out the resonant and off-resonant transmission through a sample of spatially varying thickness quasi simultaneously: As sample for this application we use a thin flake from the dichalcogenide Fe_3_GeTe_2_ exfoliated from a bulk sample and mounted on a Cu-mesh (Fig. 3a, for details see Supplementary Fig. S5). As can be seen from the photograph with the sample backlit (upper right), because of the exfoliation process, the sample itself has different thicknesses. In a resonant transmission experiment aiming at x-ray spectroscopy from this sample, the transmission at a given sample spot and x-ray energy depends on the electronic properties in that spot as reflected in the x-ray absorption spectrum but also on the sample thickness. Spectral changes mostly affect the energy region of the resonant absorption lines while thickness changes affect the whole spectrum. With two x-ray energies we are now able to separate the two contributions. We use one x-ray signal from the pre-absorption-edge region to determine the thickness variations across the sample and a second x-ray signal at the *L*_3_ absorption line to do spectroscopy.

To this end, we scanned sample across the x-ray beams and sorted the signals from the different x-ray colors by their arrival times on the detector (Fig. 2a). In this way we obtained three spatial maps, out of which we used those two with maximum energy difference. For a monochromator energy of 709 eV one signal is recorded near the absorption maximum and the other at the pre-edge (dashed line in Fig. 3c). A sample map showing only the resonant absorption distribution obtained in this way is presented in Fig. 3b. The procedure is described in the Supplementary Note 2 and Supplementary Fig. S6-10: we correct for the horizontal offset and then use the pre-edge signal to determine the thickness contribution and normalize that out of the spectroscopic signal at the absorption maximum.

We expect this particular sample to be homogeneous, so the spectroscopic changes should vanish when properly normalized. Figure 3d shows a vertical line cut at the position marked in Fig. 3b: The red curve is the raw signal at the photon energy of the absorption maximum. Plotted is the absorption, which shows a huge peak at around z = 0.75 mm and other pronounced maxima at the sample edges near z = 0 mm and z = 1.8 mm. When using the pre-edge signal to normalize out thickness variations the scan becomes considerably more flat (green curve in Fig. 3d) showing the overall success of the approach.

The normalization is probably not perfect and leaves, e.g., some residual of the strong peak in the raw signal around z = 0.75 mm behind. We assign this for the way we corrected to the horizontal offset, particularly since the offset was not commensurate with the horizontal step size, which required interpolation. Still, this example illustrates the potential of the technique especially for a horizontally dispersing monochromator that ensures that all colors hit the same sample spot quasi simultaneously.

## Conclusions

We have shown in our proof-of-principle experiment using near-edge spectroscopy in the X-ray range that the combination of transverse offset of the source points in an electron storage ring and direct imaging monochromators (e.g. PGMs) allow tunable energy shifts of a few eV at ~ 700 eV. Since the transverse position of the source point at a beamline always changes turn-by-turn when the storage ring is operated in TRIBs mode, the sample behind the exit slit is illuminated with a different color every turn (800 ns at BESSY II) at very high transverse pointing fidelity. Different source spots defined by the special static TRIBs orbit closing after three revolutions enable one to normalize resonant absorption signals on the MHz scale to, e.g., the pre-edge intensity, by simultaneous signal acquisition of successive turns and thus to directly measure true resonant absorption signals in imaging methods for chemical mapping. Since our monochromator at a 3rd generation Synchrotron Radiation source has vertical dispersion planes to adapt to the flat elliptical source, we shifted the source points vertically for our proof-of-principle experiment. With the help of a monochromator with horizontal dispersion plane and at a "round" source point expected from new 4th generation facilities, however, arrangements for such TRIBs applications will promise new opportunities (beyond fast polarization switching^12^) for spectro-microscopic techniques with unprecedented elemental contrast and sensitivity as well as much faster data acquisition.

## Methods

### Accelerator and TRIBS

**Figure 4:**
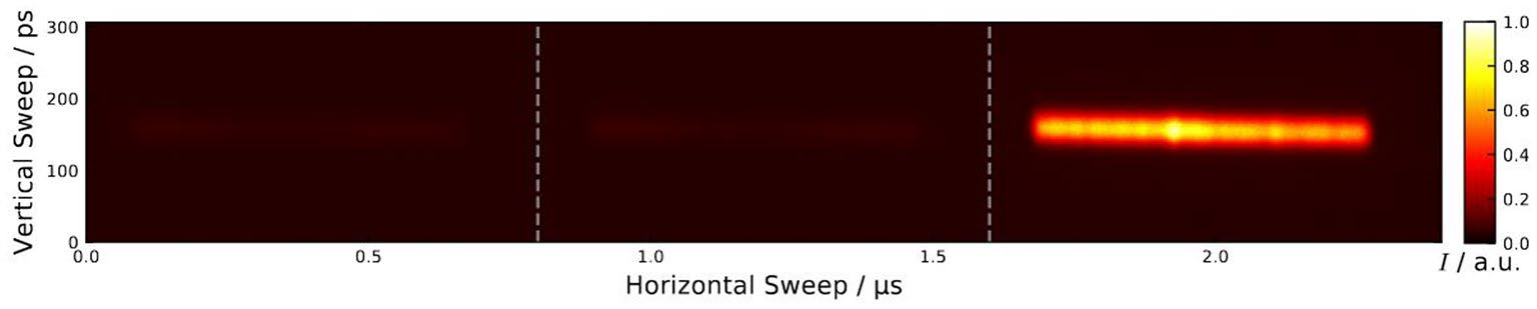
Images from the BESSY II streak camera^10^ during the experiments demonstrating single island population of TRIBs. Here only rays in the visible from one of the three source points was accepted by the optics leading to a streak at only one of the three turns (here the third one) at a purity of 94% as demonstrated by the very dark background signals from the other two previous turns (< 1.6 µs). The vertical width of the streak corresponds to the bunch length of 50 ps [FWHM] averaged over all bunches in the filling pattern.

The two-color experiment is based on a turn-by-turn displacement of source spots at the position of a beamline. Therefore, the TRIBs^2^ setting at BESSY II^3–5^ has been used, which generates a special static island orbit in the storage ring, which is winding around the reference/main orbit in the horizontal plane, closing after three revolutions. Depending on the storage ring setting, both orbits (island and main) can exist in parallel and store different electron beams (see inset (d) in Fig. 1), but also only the island orbit alone can be populated with electrons, as it was used for this experiment. The displacement of the source spots depends on the horizontal working point (tune) of the storage ring and its distance towards the 3rd order resonance as well as the tune-shift-with-amplitude are defined by non-linear sextupole magnets. The standard orbit closing after one revolution can store up to 400 electron bunches at BESSY II. The island orbit, due to the three folded length can store up to 1200 electron bunches. If only, say, the third 400 buckets are filled then, only light from one source spot in the third turn is emitted and seen by a beamline as proven by Fig. 4, where only one out of the three source points was accepted by the streak camera^10^.

This single-island-population (SIP) was achieved by non-linear resonant excitation as described in detail in Ref.^3^. For the proof-of-principle experiment described here, the ring was filled with 300 out of 1200 (400 for the main orbit) buckets at an injected ring current of I = 50 mA in decay mode with a lifetime of ~ 15 h. A good SIP of about 94% purity was reached with 3.2 and 2.8% residual charge captured in the other two island buckets as quantitatively derived from the streak camera data in Fig. 4.

For a well decoupled storage ring, the three island spots on a source point imaging system appear only displaced in the horizontal plane and the vertical displacement is negligible as shown in inset (c) of Fig. 1. The skew-quadrupoles have been used to introduce a vertical separation of the island spots as required for our experiments and to put bunches from successive turns vertically over each other at a selected source point (inset (d) of Fig. 1). Displacements of up to 10 mm at all available source points, including the straight sections along the electron closed orbit are possible this way.

During the past few years we have provided special dedicated user weeks of BESSY III in TRIBs mode to figure out if a potential second orbit wit single bunch population in regular ToUp mode is feasible. The result was that is indeed possible to tweak most of the beamlines in a way to either accept the second or the regular orbit. If only a part of the fill pattern (e.g. the camshaft bunches for time-resolved studies) are kicked to the TRIBs orbit, these bunches can be inhibited by gate pulses if fast detectors are used. However, there are users with slow detectors being only interested in CW operation at highest performance without gating capabilities who might feel disturbed. Hence, we are aiming at dedicated user weeks in TRIBs mode e.g. with all bunches in TRIBs orbit, in the same manner we are operating the so-called low-a operation (2 weeks/year) or single bunch mode (4 weeks/year) for users with interest in special beam properties. If the TRIBs approach will turn out to be beneficial for most users, even a standard operation can be envisaged, especially at our 4th generation successor BESSY III, where the separation of point like sources in all beamlines will be easier and can be planned from the beginning.

### Monochromator and experiment

The experiments were performed in the scattering chamber behind the PM3 monochromator^7^ located on bending magnet DIP112 at section H11 at the BESSY II storage ring. The monochromator is a collimating plane grating monochromator^8,9^ (PGM), a successor of the famous SX700 type, that images the source point vertically de-magnified onto the exit slit and horizontally 1:1 onto the sample (see the optical layout in Supplementary Fig. S2) which is part of our conceptual approach, namely that photons from vertically separated source points in the storage ring hit the sample behind the slit at different energies. As a usual monochromator setting we used the G = 1221 l/mm grating at c_ff_ = 2.25 (we tested also c_ff_ = 1.4…12), 100 µm exit slit and circular polarization (S =  + 0.4)^7^. The grating can be easily changed to 600 l/mm at the PM3 using stepping motor controls. At other similar monochromators up to 3 gratings from 150 to 2400 l/mm are routinely available to change the line density within few seconds.

As detector we employed a windowless Avalanche Photodiode (APD) of 3 mm active area from Hamamatsu S8664-30 K (140 MHz bandwidth). The trigger was the 1/3 sub-harmonic (416 kHz) of the 1.25 MHz revolution trigger derived by a freely programmable gate array (FPGA) based divider (BMESG08-p) from the optically distributed 500 MHz master-clock signal as gated by the 1.25 MHz bunch clock trigger. Signal acquisition was performed by (i) a LeCroy 4 Gs/s oscilloscope and (ii) a Zürich Instruments UHFLI lock-in amplifier with a twofold digital boxcar feature, respectively. In case of (i) the signal was averaged in sequence mode over 3 identical gate windows covering the macro-pulse signal from the full fill pattern consisting of 300 bunches at 200 ns dark gap, the latter used as reference signal for baseline detection. The UHFLI (ii) boxcar turned out as a good pick for the two-color imaging applications at excellent signal-to-noise using 4 gate windows for signal and baseline signal from successive turns, respectively.

For the spectral results in Fig. 2 we used a transmission sample of 15 nm Fe on Si_3_N_4_, which shows a 50% absorption dip at the Fe L_3_ edge. The sample for the scanning imaging example from Fig. 3 was a thin freestanding film of the dichalcogenide magnetic 2D material Fe_3_GeTe_2_^11^ (see Supplementary Fig. S5) of variable thickness on a copper mesh leading to an average resonant absorption of likewise ca. 50% at the Fe L_3_ edge. The material, a van der Waals magnet^11^, is of relevance for potential voltage-controlled magneto-electronics but used here as a challenging case to demonstrate our new two-color method that allows for separating thickness contrast from resonant absorption and a potentially much higher contrast by MHz normalization in microscopy applications.

## Supplementary Information

Supplementary Information.

## Data Availability

The data that support the findings of this study are available from the corresponding author upon reasonable request.
