# Supplementary material for: Two-color synchrotron X-ray spectroscopy based on transverse resonance island buckets
Source: Sci Rep. 2022 Sep 1;12:14876. doi: 10.1038/s41598-022-19100-z (PMC9437009; doi:10.1038/s41598-022-19100-z)
Supplement: Supplementary file 1 — Supplementary Information. [file 41598_2022_19100_MOESM1_ESM.docx]

**Supplementary Material: Two Color synchrotron X-ray spectroscopy based on transverse resonance island buckets**

K. Holldack^1^, C. Schüßler-Langeheine^1^, N. Pontius^1^, T. Kachel^1^, P. Baumgärtel^1^,

Y.W. Windsor^2^, D. Zahn^2^,

P. Goslawski^1^, M. Koopmans^1^ and M. Ries^1^

^1^Helmholtz-Zentrum Berlin für Materialien und Energie GmbH. Albert-Einstein-Str. 15, D-12489, Berlin, Germany

^2^Fritz Haber Institute of the Max Planck Society, Faradayweg 4-6, D-14195 Berlin, Germany

**Supplementary Note 1**

**Derivation of the dispersion formula**

Using the grazing incidence notation of the grating equation α und β > 0 from H. Petersen (1981)^1^ one starts with

$$=\frac{d}{m}\left( \cos\left( \right)-\cos\left( \right) \right). (1)$$

Where d is the groove spacing and m the diffraction order of the grating. In the collimated PGM^2^ design by R. Follath, the M1 mirror tilts the parallel beam onto the plane mirror M2 by d = dy/F and F is the focal length of M1 (vertical arm length of a toroid, F=13 m).

The complete differential from (1) yields:

$$d=\frac{d}{m}(sin d-sin d) (2)$$

To get the beams through the exit slit the re-collimating optics requires d$\beta=0$ (see Figure 1 in the main manuscript) because beams at different deflection angles do not pass the slit, hence it remains:

$$\frac{d\lambda}{d}=\frac{d}{m}\sin(3)$$

For the photon energy it follows with Ε as given by $=hc/$ *d*$\alpha$ *= d* (see above):

$$d=\frac{d}{mhc}\sin\alpha{}^{2}d\gamma(4)$$

With abbreviations: groove density $G=\frac{1}{d}$ , $HG=hcG$ und $m=1$ (first diffraction order) one gets:

$d=\frac{{}^{2}}{\mathrm{HG}}\sin\alpha d\gamma$ (5)

For G = 1221 l/mm, HG = 1.51385 eV and finally inserting the change of the grazing angle on the pre-mirror $d\gamma= dy/F$ one ends at:

$\frac{d}{dy}=\frac{{}^{2}}{GHF}\sin\alpha$ (6)

Or with numerical values at *F* = 13 m:

$\frac{d}{dy}=\frac{\sin\alpha{}^{2}}{19680} [\frac{eV}{mm}]$ (7)

The grazing angle α on the grating follows from the backward grating equation vs. *E, HG* and *c_ff_* with the definitions above:

$\alpha\left( ,c_{ff},HG \right)= \cos^{-1} \left( \frac{HG}{\left( 1-{c_{ff}}^{2} \right)}+\sqrt{1+\left( \frac{c_{ff}HG}{\left( {c_{ff}}^{2}-1 \right)} \right)^{2}} \right)$ (8)

In addition to the analytical estimation, we have performed a full raytracing using the code Ray-UI^3^ with all parameters from the PM3 monochromator. As revealed by Table S1 and Table S2, there is excellent agreement between both approaches after we recognized that the assumption to set Δβ = 0 is key for the analytical simulation. This condition is obvious because only rays fulfilling it may pass the exit slit. Rays arriving with the same energy from displaced spots cannot pass it since they are offset along the dispersion plane.


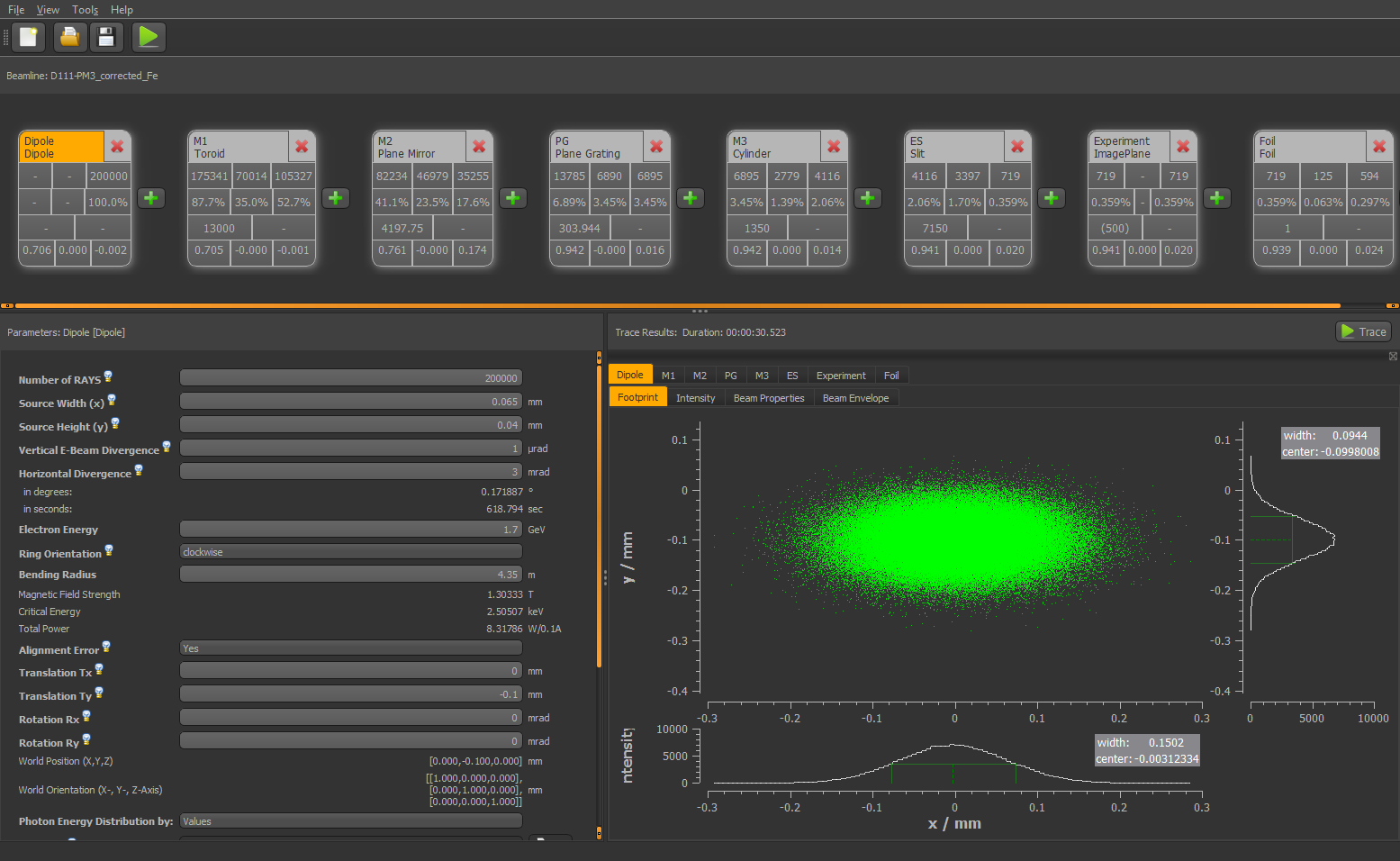

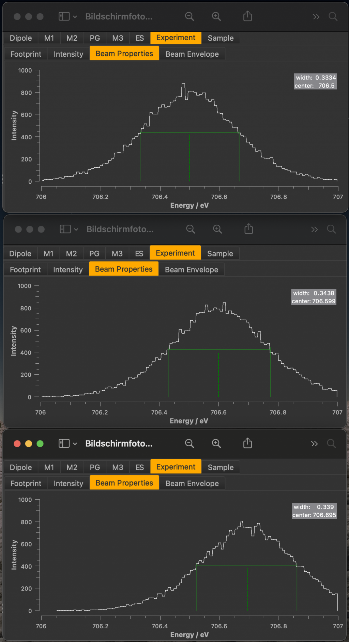


**Fig. S1:** Screenshot from the RAY-UI^3^ simulation user interface displaying the plot of the simulated BESSY II dipole source as displaced by 0.1 mm (left) and the energy shift of rays behind the exit slit for an assumed monochromatic source at vertical displacements from -0.1, 0 and +0.1 mm at c_ff_ =2.25. The photon energy shifts at these settings by 0.8 eV/mm.

**Comparison of simulation results**

**Table S1:** Calculations of the dispersion at the PM3 for different energies and c_ff_ values using equ. (6), error bars are 0.01 eV/mm.

| **c_ff_-Factor** | **dE/dy@400 eV** | **dE/dy@700 eV** |
| --- | --- | --- |
| 1.4 | 0.72 eV/mm | 1.67 eV/mm |
| 2.25 | 0.35 eV/mm | 0.811 eV/mm |

**Table S2:** Corresponding raytracing results from RAY-UI, error bars are 0.01 eV/mm.

| **c_ff_-Factor** | **dE/dy@400 eV** | **dE/dy@700 eV** |
| --- | --- | --- |
| 1.4 | 0.71 eV/mm | 1.65 eV/mm |
| 2.25 | 0.35 eV/mm | 0.8 eV/mm |

The behavior of the photon beam emitted from offset source points can be considered as understood. Although this was calculated for the collimated PGM, the basic principle is generally valid also for other monochromators since the cause is mainly the lattice equation.


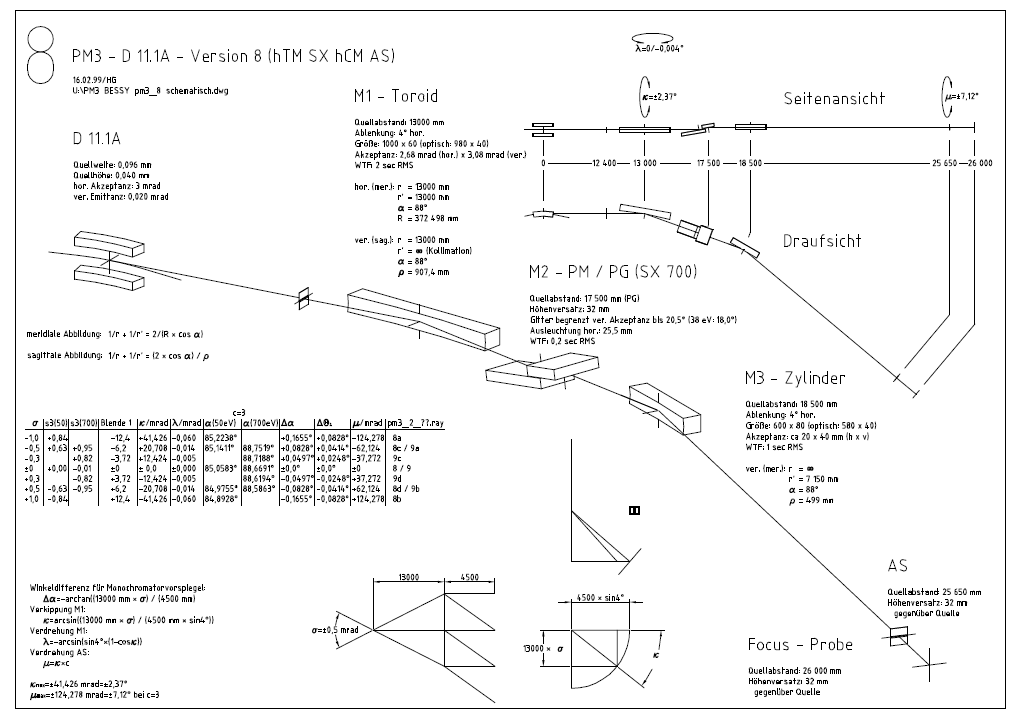


**Fig. S2:** Layout of the PM3 Monochromator used in the experiments, a collimating plane grating monochromator of the SX700 type^1,2^.


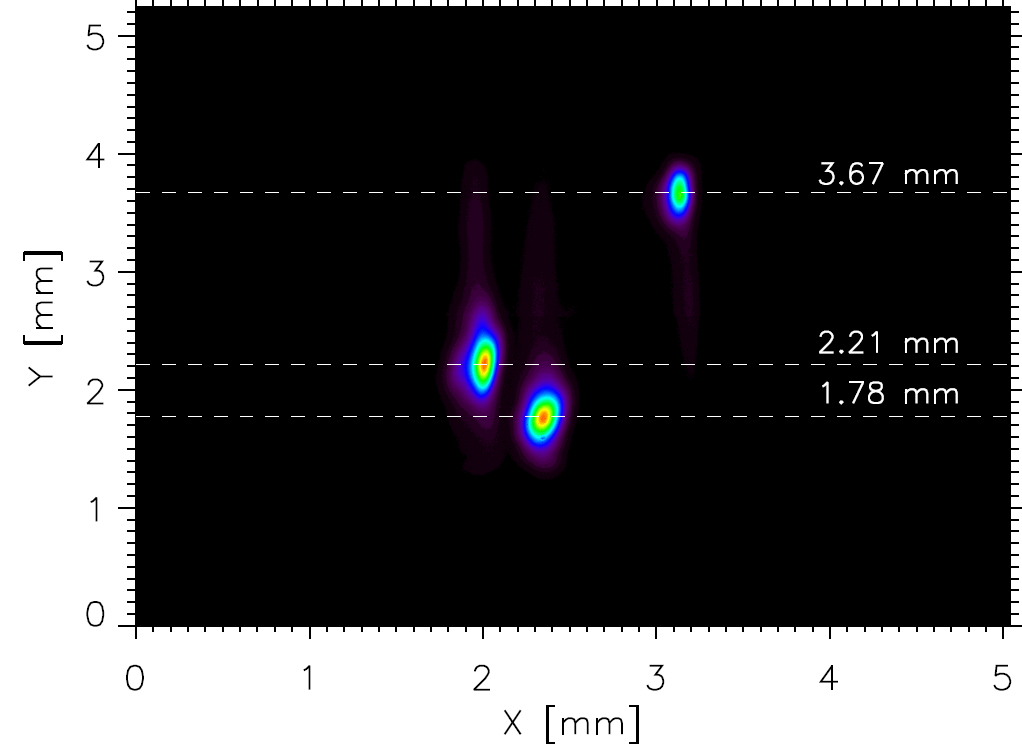


**Fig. S3:** Source point distances measured with a visible light imaging system at an equivalent dipole source point in the storage ring section after the PM3 for the accelerator settings used in the experiments. The right upper spot appears weaker owing to intensity loss given by the limited acceptance of the optics.


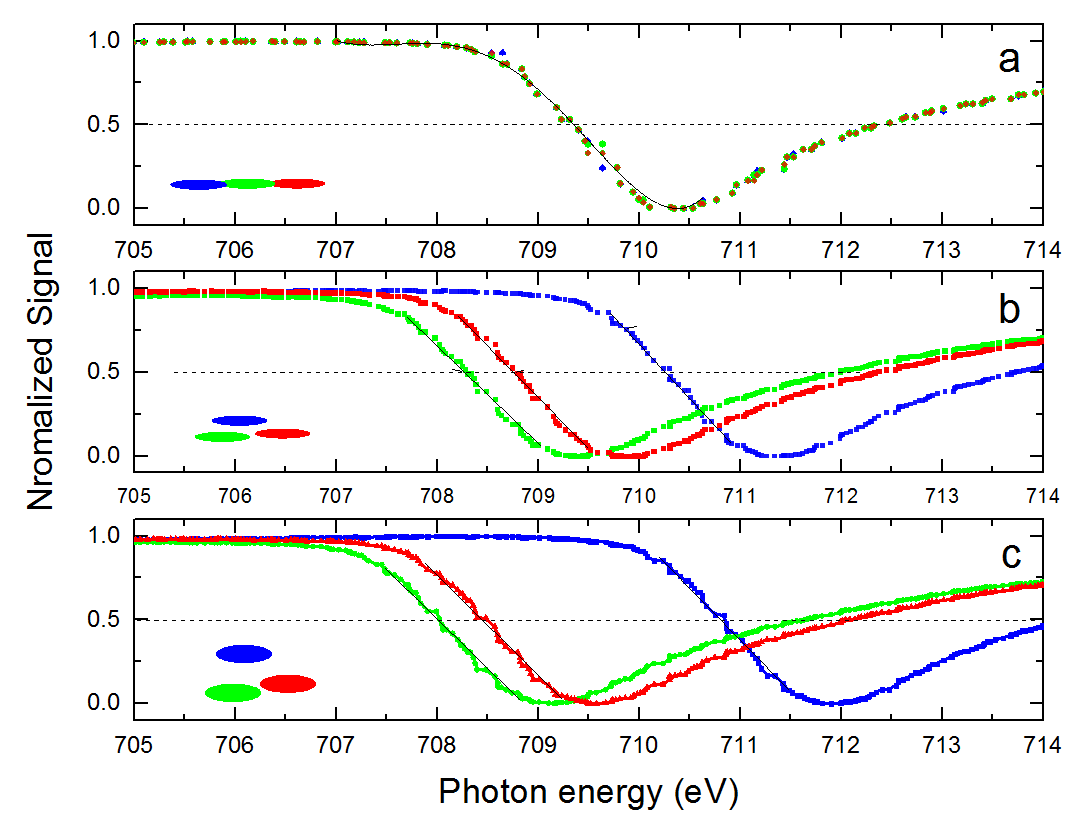


**Fig. S4**: Spectra from all three macro-pulses from the 1^st^ (blue), 2^nd^ (red) and 3^rd^ (green) turn for 3 different settings of the storage ring parameters. (a) decoupled, (b) Sep2 setting and (c) Sep3 setting. The energy shift follows the vertical separation of the three source points, best agreement is with measured spot separations at the diagnostic station on a dipole closest to the PM3 source.

| **Acc-mode**  **and detectors** | **1st** | **2nd** | **3rd** | **Maxdiff**  **(eV)** | ***dE/dy***  **measurement**  **(eV/mm)** | ***dE/dy***  **equ. 1**  **c_ff_=2.25**  **(eV/mm)** | ***dE/dy***  **RAY**  **c_ff_=2.25**  **(eV/mm)** |
| --- | --- | --- | --- | --- | --- | --- | --- |
| **Spectral**  **Shift** | eV | eV | eV | eV |  |  |  |
| *Sep2* | 708.79 | 710.23 | 708.23 | 2.00 |  |  |  |
| *Sep3* | 708.44 | 710.74 | 708.02 | 2.72 |  |  |  |
| ***Spots-PINH03*** | µm | µm | µm | µm |  |  |  |
| *Sep2* | 524 | -954 | 138 | 1478 | 1.35 | 0.811 | 0.8 |
| *Sep3* | 847 | -1394 | 229 | 2241 | 1.21 | 0.811 | 0.8 |
| ***Spots-DIAG*** | µm | µm | µm | µm |  |  |  |
| *Sep2* | 2210 | 3670 | 1780 | 1890 | **1.06** | **0.811** | **0.8** |

**Table S3:** Source displacements, energy shifts and measured dE/dy compared to predicted dispersion values. Statistical error bars for the source positions are ± 10 µm and ± 0.01 eV/mm for the simulations. The masuremnts are somewhat larger since both simulations ignore y’, a vertical angular change of the electron beam at consecutive turns.

**Supplementary Note 2**


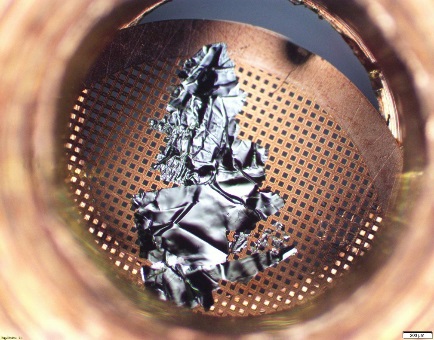

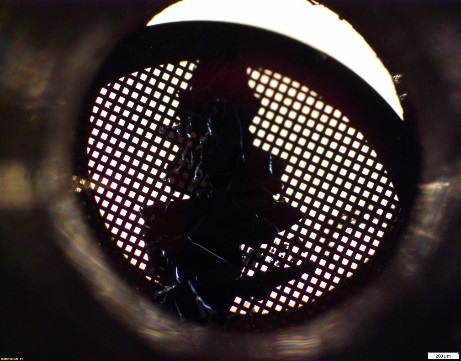

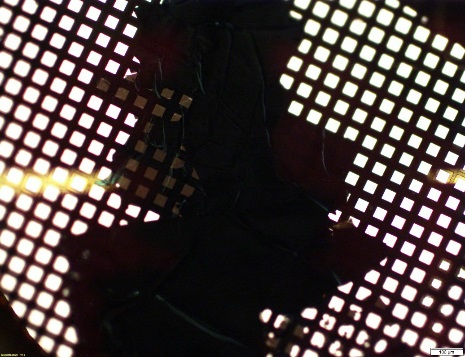


**Fig. S5:** Optical images of the freely standing sample of Fe_3_GeTe_2_. (left) microscope image in reflection, (middle) transmission image and (right) a close-up. The film thickness is not homogeneously distributed and is hard to be corrected by conventional normalization methods since an “empty case” is missing and a real *I_0_* signal is lacking as it would need an identical measurement of the cleaned mesh. Courtesy: W. Windsor, D. Zahn, L. Rettig, Fritz-Haber-Institute Berlin (FHI).

**Analysis of the 2-color transmission map**

For the analysis of the sample from Fig. S5 we used the signals from turn #1 and turn #3 which have the largest relative energy offset. Fig. S6 shows an energy scan of the transmission through the Fe_3_GeTe_2_-sample from Fig. S6 for the two signals from consecutive turns. We set the monochromator to the nominal energy of 709.3 eV (vertical line), which corresponds to the absorption maximum for turn #3 and the pre-edge transmission for turn #1. In the following we will refer to these as on-resonance and off-resonance signals, respectively.


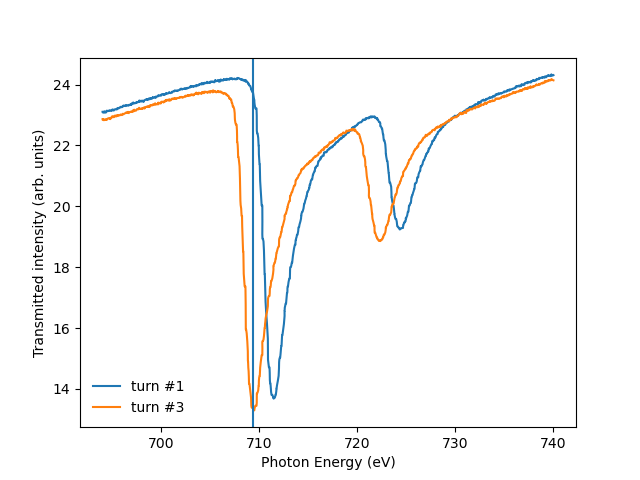


**Fig. S6**: Intensity transmitted through the Fe_3_GeTe_2_ sample from Fig. S5 measured from the macropulses of the consecutive turns #3 and #1.

For our demonstration with a vertically dispersing monochromator, the energy shift also implies a horizontal offset of the beam on the sample. Fig. S7 shows spatial maps of the transmitted signal for the two different signals.

| 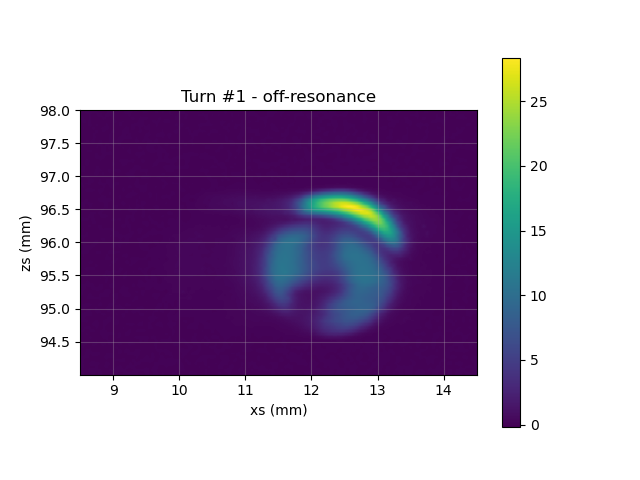 | 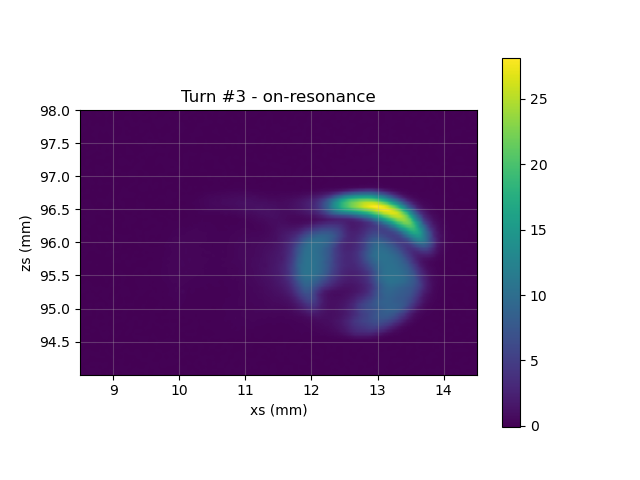 |
| --- | --- |

**Fig. S7:** Scanning transmission images simultaneously taken with the macro-pulses from subsequent turns (Turn #1 and Turn #3). Since the two source points involved at this machine setting, they appear horizontally shifted in “xs”.

We determined the spatial offset by cross-correlating the two maps from Fig. S7. Since the offset of 0.41 mm was incommensurate with the step size of the maps (0.05 mm), we linearly interpolated the signal along the horizontal direction to a five-time higher point density (point distance 0.01 mm). As expected for the monochromator optics, we found no noticeable vertical offset so there was no need to interpolate the data along this direction.

| 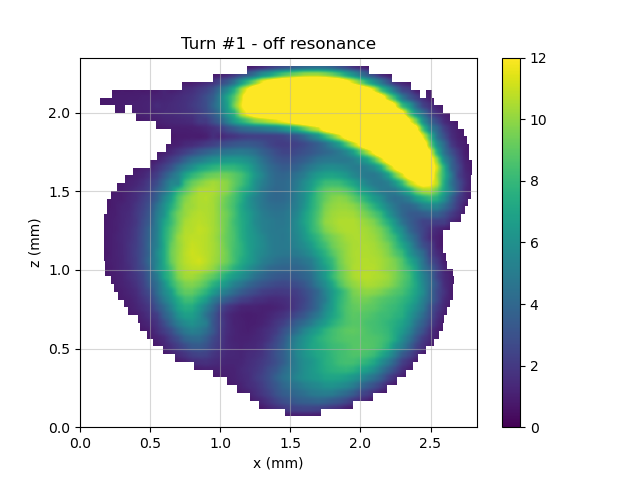 | 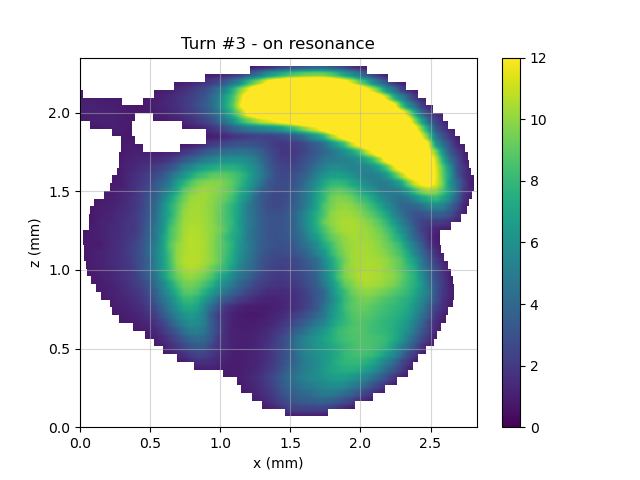 |
| --- | --- |

**Figure S8:** The two maps from turn #1 (left) and #3 (right) aligned on a common spatial scale.

Fig. S8 shows the two aligned maps on a common spatial scale. The region of the sample holder with zero transmission (signal level < 0.8) was masked out to avoid numerical problems. The sample is visible as a darker region around x = 1.5 mm. The inhomogeneous signal in the off-resonance signal shows the variation in sample thickness. (The false-color-scale is set to cover the region of the sample; the colors in the crescent-shaped high-intensity region at the top caused by the opening in the sample holder without any absorbing structure in the X-ray beam are saturated.)

Near electronic resonances, the absorption coefficient depends critically on the electronic and magnetic properties of the sample in the probed sample region. In a transmission experiment, the resonant transmission, $T_{R}$, is therefore expected to depend on the local sample absorption coefficient $\mu(x,y)$ and on the local sample thickness $d\left( x,y \right)$.

$$T_{R}=\exp(-\mu\left( x,y \right)d\left( x,y \right))$$

For a sample of inhomogeneous thickness, both $\mu(x,y)$ and $d\left( x,y \right)$ vary spatially. At energies off resonance, the sensitivity of the absorption coefficient on local electronic properties is very weak. We can therefore assume the non-resonant transmission, $T_{N}$, to vary only due to changes in the local thickness with a constant absorption coefficient $\mu_{0}$:

$$T_{N}=\exp(-\mu_{0}d\left( x,y \right))$$

We can now normalize the thickness contribution out when taking the ratio of the logarithms of the non-resonant and resonant transmissions and obtain the local absorption coefficient relative to the non-resonant absorption coefficient:

$$\frac{\mu\left( x,y \right)}{\mu_{0}}=\log T_{N}/\log T_{R}$$

| 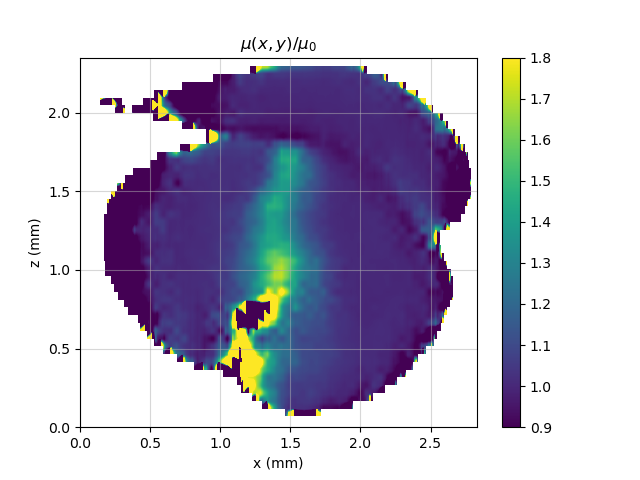 | 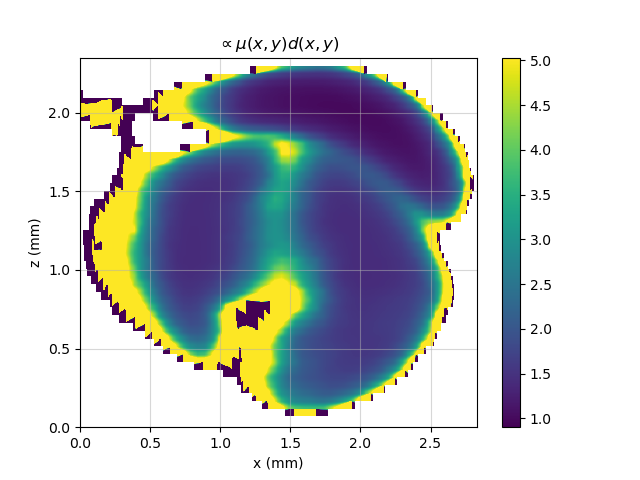 |
| --- | --- |

**Fig. S9:** Comparison of the normalized (a) and non-normalized map (b).

Figure S9a shows the map normalized in this way. For comparison Fig. S9b still contains the non-normalized resonant absorption.

| 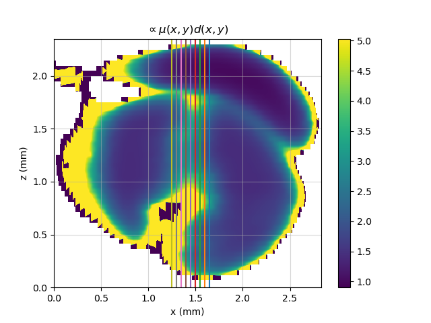 | 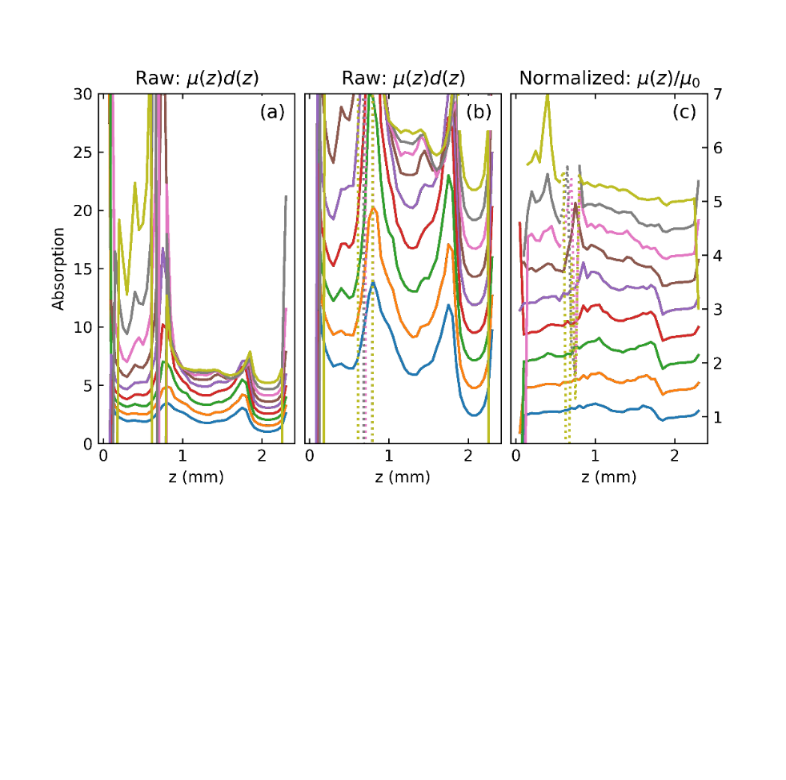 |
| --- | --- |

**Fig. S10:** Non-normalized map (left) and corresponding line cuts through the sample region for the normalized (right, a,b) and non-normalized cases (right, c) as centered at the positions marked by the colored vertical lines in the map on the left.

Figure S10 a,b,c shows line cuts through the sample region centered at the positions marked by the colored vertical lines in the map on the left. The strong absorption modulations found in the raw transmission map [shown in (a) on an extended vertical scale and in a vertically magnified view in (b)] are due to thickness variations and mostly normalize out nicely as the line cuts in (c) show. The normalization only breaks down in the very thick sample region near x = 0.8 [yellow region in the map, dotted lines in (b) and (c)].

**Supplementary References**

1. Petersen, H. The plane grating and elliptical mirror: A new optical configuration for monochromators, *Optics Comm.* **40** (6), 402-406, (1982).
2. Follath, R., The versatility of collimated plane grating monochromators, *Nucl. Instr. & Meth. A* **467-468**, 418-425 (2001).
3. [P. Baumgärtel, P. Grundmann, T. Zeschke, A. Erko, J. Viefhaus, F. Schäfers, and H. Schirmacher, RAY-UI: New Features and Extensions, *AIP Con. Proc*. **2054**, 060034 (2019)](https://aip.scitation.org/doi/abs/10.1063/1.5084665).
